# Is India ready for mental health apps (MHApps)? A quantitative-qualitative exploration of caregivers’ perspective on smartphone-based solutions for managing severe mental illnesses in low resource settings

**DOI:** 10.1371/journal.pone.0203353

**Published:** 2018-09-19

**Authors:** Koushik Sinha Deb, Anupriya Tuli, Mamta Sood, Rakesh Chadda, Rohit Verma, Saurabh Kumar, Ragul Ganesh, Pushpendra Singh

**Affiliations:** 1 Department of Psychiatry, AIIMS, New Delhi, India; 2 Department of Computer Science, IIIT-Delhi, Delhi, India; University of Birmingham, UNITED KINGDOM

## Abstract

**Background:**

Mobile application based delivery of psycho-social interventions may help reduce the treatment gap for severe mental illnesses (SMIs) and decrease the burden on caregivers. Apps developed in high income settings show effectiveness, but they suffer from lack of applicability in low resource scenarios due to the difference in technology penetration, affordability, and acceptance.

**Objective:**

This study aimed to understand health technology usage, perceived needs, and acceptability of app based interventions in patients with SMIs to improve illness management and reduce caregiver burden.

**Methods:**

The study was conducted in inpatient and outpatient settings of a tertiary care center in North India. A cross-sectional survey assessed smartphone and health app usage. Further, three focus group discussions evaluated the needs and apprehensions in using apps in management of SMIs.

**Results:**

A total of 176 participants including 88 patients and 88 caregivers completed the survey. Smartphone ownership was similar to the national average (30%) in both caregivers (38.6%) and in patients (31.8%). Although subjects regularly used a third party app, health app usage was very low. Cost, unfamiliarity, and language were significant barriers to adoption. The focus group discussions provided insight into the various apprehensions of caregivers in using and in allowing patients to use smartphones and such apps. Caregivers wanted mobile apps for accessing information regarding services and resources available for people with SMI, and they felt such apps can be helpful if they could automate some of their routine caregiving activities. However, the significant difficulty was perceived in regards to the cost of the device, language of the medium, and unfamiliarity in using technology. Apprehensions that SMI patients might misuse technology, or damage the device were also prevalent.

**Conclusions:**

The study systematically looks into the scope, design considerations and limitations of implementing a mobile technology based intervention for low resource settings. With only one-third of the patients and caregivers having access to smartphones and internet, parallel outreach strategies like IVRS should be actively considered while designing interventions. The difficulty of understanding and searching in a non-native language needs to be addressed. Hand holding of caregivers and frequent encouragement from treating doctors might significantly help in technology adoption and in surmounting the apprehensions related to using technology. To make the solution acceptable and useful to the already over-burdened caregivers, developers need to work closely with patients’ family members and follow a ground-up collaborative approach to app development. The scope of delivering mental health services through technology is immense in resource constrained settings like India, provided we, researchers, appreciate and accept the fact that in the varied landscape of a divergent economic, educational, and cultural milieu, a single solution will never suffice for all, and intervention modality matching with end user capacity will be of paramount importance in determining the success of the endeavor.

## Introduction

Severe mental illnesses (SMIs) are disabling, chronic psychiatric conditions that affect individuals in their prime and extol substantial burden on patients and their caregivers [1]. Schizophrenia and bipolar disorders, the two major SMIs far surpass most physical disorders in terms of illness cost and disability [2–4]. In addition to pharmacotherapeutics, psycho-social interventions geared towards health literacy, medication adherence, self-monitoring and vocational support have been shown to help such patients live productively and independently [5]. In resource constrained settings of low and middle income countries (LAMI), such interventions are mostly provided through the family members of the patient, who, therefore, become an invaluable asset to bridging the treatment gap [6]. Literature shows that the potential for a patient’s family in facilitating recovery has generally been under recognized [7–9]. To help caregivers in their support activities and to provide psychosocial interventions to patients, technology based interventions, as a group, posit a practicable and effective alternative [10]. In this paper, we interchangeably use the terms family members and caregivers to denote informal caregivers. We acknowledge that not all family members are caregivers, nor are all the caregivers informal caregivers. We request that our paper be read as such.

A review of existing literature shows a heightening of interest in the delivery of technology based interventions over the last decade with the focus gradually shifting from computer based to internet based and finally to mobile based interventions as technology permeates more into our lives and becomes affordable. Mobile based solutions can potentially reduce the burden of health systems, empower patients and break barriers to help-seeking [11]. Smartphones (mobile phones with capacity to install third party apps), additionally, have numerous built-in sensors, which can be used for the real-time ecological momentary assessment (EMA) [12, 13] of the patient’s symptoms. The current World Health Organization (WHO) policy, therefore, also suggests devising processes for self-care in health by the use of electronic and mobile devices, especially in resource limited settings [14]. Globally, mental health apps (MHApps) have been used for psychosis, [15, 16] bipolar disorder [17], depression [18], suicide [19], anxiety [20], eating disorders [21] and substance use [22, 23], as well as for improving service delivery [11] and monitoring program outcome [24] in the field of psychiatry. However, MHApp represents an umbrella term incorporating diverse types of apps that differ in their mode of intervention delivery, mobile platforms and the capability of the mobile devices in terms of monitoring sensors and internet connectivity, thereby limiting comparability [11, 25–27]. Critics have also argued that many MHApps rely on the novelty of their modus operandi rather than on technology acceptance measures and behavior change theories [28], which limit the long-term usefulness of such apps. Additionally, app usage suffer from lack of patient’s interest and willingness to engage because of the challenges in using technology, and even simple SMS reminders for medication adherence may go unused by patients [29, 30]. Lack of stakeholder’s participation at the development phase of the app leads to limiting the design usefulness for the end-user [31]. Technology expertise mismatch between developers and end-users often result in non-optimal app experiences [32]. Finally, the interventions delivered through the apps need to be topically relevant, and acceptable to the patient and caregivers, thereby rendering such apps to be locally effective rather than universally usable. It is therefore of vital importance to understand the factors, which drive engagement and disengagement of end-users with mHealth technologies [33]. In India, mental health resources for psychosocial interventions are meager [34], and greater than 80% of patients suffering from SMIs fail to receive minimally adequate treatment for their illness [35, 36], creating a mental health treatment gap of more than 80%. At the same time, India is going through a digital revolution with its telecommunication network currently reported as the second largest in the world. At present, 73.34% of the country’s population is connected by phones, 96.6% of which are wireless mobile phones [37]. The Internet penetration in the country is currently 34.8%, and shows a steady growth in adoption in cities as well as in the rural areas [37]. However, utilization of these digital resources in the field of health care in general and mental health care in particular are very low. Literature shows only a handful of projects that deliver mobile based services for health information [38, 39], HIV [40, 41], diabetes [42], tuberculosis [43], cardiovascular diseasesÂ [44, 45] in India, and only two SMS services for mental health issues [46, 47]. To the best of our knowledge, no research from India highlights the use of app based intervention for mental disorders. The absence of technology based interventions in the field of mental health in India forces us to evaluate the problems related to developing solutions which are effective, user acceptable, clinically relevant, and locally deployable in our low resource setting. The overarching aim of this study therefore is to understand how mental health patients and their caregivers use digital technology in relation to their mental health and caregiving needs and what new design possibilities exist in improving available health care services. As a primary objective, our research endeavored to understand the technology usage capability and health technology usage by patients suffering from severe mental illnesses and by their caregivers. Further, we intended to study the perceived needs of the caregivers for technology based health solutions and explore their apprehensions and roadblocks in using such technology through focus group discussions (FGDs). In this work, we extend our preliminarily study—result of first FGD—in Mental Health: Sensing and Intervention (Ubicomp 2016 workshop) [48]. Working towards our objective, we surveyed 88 patient-caregiver pairs and conducted three FGDs (including the first FGD presented in [48]) with caregivers.

Such a quantitative-qualitative mixed methodology approach provides us with an in-depth understanding of the factors that bear significance in the development and deployment of mobile technology based solutions in the Indian population. Additionally, while most MHApps from high-income countries focus on self-usage and self-management by patients, in keeping with literature review, we sought to understand the interest in mobile-based psychosocial interventions and acceptance among patient caregivers.

## Methodology

A research collaboration was formed between clinicians from our tertiary care center, India Institute of Medical Sciences (AIIMS), New Delhi, India, and experts in the Human Computer Interaction (HCI) group from the Indraprastha Institute of Information Technology, Delhi, to understand the capabilities and limits of a mobile technology platform in delivering services to cope up with severe mental illnesses. Multiple rounds of discussions were conducted to make the computer science experts understand the principles of management of severe mental illnesses while making us aware of various mobile technology platforms and possibilities. To achieve our objectives of technology usage and health technology needs in patients and their caregivers, a cross-sectional quantitative and qualitative mixed method protocol was adopted [49]. We administered a survey and conducted a series of focus group discussions with the caregivers’ of patients with SMIs. The study was conducted at the psychiatry outpatient and inpatient facility of our tertiary care center at New Delhi from February 2016 until March 2017 and was approved by the Institute Ethics Committee, AIIMS, New Delhi, India. The following subsections present each of our methods in detail.

### Participants and inclusion criteria

For the purpose of the quantitative survey, the participants for the study were selected using a purposive consecutive sampling method [50]. All patients suffering from SMI, aged 18 to 60 years, admitted in the psychiatry ward or attending the follow-up outpatient SMI clinic, between February 2016 and December 2016, were screened. The primary caregiver of these screened patients, who were themselves not suffering from any mental disorder or cognitive disability, were invited to participate in the study. Written, informed consent to participate in the study was collected from the willing caregivers after explaining to them the details of the study. Information regarding the patients’ treatment and mobile use was provided primarily by the caregivers. Additionally, patients themselves provided written assent to share their illness and treatment details in the same consent form under supervision of their caregivers and often provided additional details regarding their mobile phone usage. Anticipating possible communication difficulty in patients suffering from SMIs, the questionnaire was designed and worded to be filled by the caregiver.

Participation in the FGDs was voluntary and followed a purposive sampling technique. The Caregivers of patients who fulfilled inclusion and exclusion criteria were invited to participate in the FGD and written informed consent was taken. At all steps, for both the methods (survey and FGDs), the study procedures were explained, and written consent was obtained at the beginning of each person’s participation in the study. The confidentiality of all participants and their patients were ensured. Inclusion in the study did not have any bearing on the patient’s outpatient treatment or on the duration of inpatient hospital stay, and participants had the right to decline any information or leave the study at any point in time.

For the purpose of the study, SMI was defined as a diagnosis of schizophrenia and other related psychosis or bipolar disorder as per the International Statistical Classification of Diseases and Related Health Problems 10th Revision (ICD-10) [51] for a duration of more than two years. The primary caregiver was defined as the person who, according to the patient, provided most of his/her support needs, was staying with the patient, and served as the primary contact for the patient’s medical needs [52]. Considering that smartphone penetration in the country is around 30% [53], a sample size of 85 in each group (patients and caregivers) was calculated as necessary at 10% precision for the survey to capture the mobile usage patterns of the target population.

### Survey

The quantitative survey focusing on mobile and health technology usage of patients of SMI and their caregivers was conducted through a self-developed, semi-structured questionnaire. The questionnaire items were developed from existing literature [54], with additional items added as per requirements of the technology experts and was modified collaboratively over the subsequent group discussions. The tool enquired into the socio-demographic and clinical profile (of patients), mobile phone use and use of health apps in both the patient and their primary caregivers. In addition, data related to the technical specifications of the current mobile phone, including operating system, sensors available in the phone, and internet connectivity were collected to help in deciding app development. The questionnaire included 49 questions, with 25 capturing technology usage, 2 targeting daily caregiving practices and challenges, 8 focusing on patient medical details, 10 exploring demographic details of caregivers, and 6 on demographic details of the patients. The tool included both open-ended (e.g., *“Common activities requiring [a caregivers’] support”*) and closed ended questions (e.g., *“Do you think such ‘apps’ will add to the burden of caregiving?”*) The tool was applied to the patients and their caregivers by a trained psychiatrist and took around 10 minutes to complete.

### Focus group discussions (FGDs)

We approached the perceived needs of the caregivers for technology based health solutions and their design scope through a series of qualitative focus group discussions. We conducted three FGDs, spanning two hours each in the in-patient ward of the hospital, inviting the caregivers of the admitted patients with diagnosed SMIs fulfilling the aforementioned inclusion and exclusion criteria.

The three FGDs included a total of 26 participants, including 15 caregivers of patients, details of which are provided in Table 1. In addition to caregivers, each FGD also included various service providers like occupational therapists, psychiatry residents, and clinical psychologists. It felt necessary to create a mixed group of stakeholders for the FGDs as patients’ caregivers often felt inhibited and unsure in front of clinicians in starting conversations focusing on their needs and problems. A mixed group often helped in initiating discussions, encouraged extensive cross-communication between caregivers and other care providers, and promoted a wider perspective to the problems faced in caregiving. For the purpose of the current study, we focus exclusively on the inputs provided by the caregivers and their perspectives.

**Table 1:** Distribution and coding of focus group (FGD) participants (n = 22, care-providers = 15).

Focus Group	Participants	Code	Relation of care-providers with patient
F1	Caregivers = 4, Occupational therapist = 1, Psychiatry Resident = 1, Clinical Psychologist = 1	[F1C1]…[F1C4]	Parents [F1C1],[F2C2], Son [F1C3], Brother [F1C4]
		[F1T1]	
		[F1R1]	
		[F1P1]	
F2	Caregivers = 6, Occupational Therapist = 1, Psychiatry Resident = 1	[F2C1]…[F2C6]	Parents [F2C1]…[F2C6]
		[F2T1]	
		[F1R1]	
F3	Caregivers = 5, Occupational Therapist = 1, Psychiatry Resident = 1	[F3C1]…[F3C5]	Parents [F3C1]…[F3C3], Spouse [F3C4], Brother [F3C5]
		[F3T1]	
		[F3R1]	

The moderators of the FGDs consisted of at least two psychiatry faculty members and one faculty member from the department of computer science. Working towards our objective, we started each interview by asking caregivers about the burdens they face. Subsequent questions focused on the caregivers’ perspectives on the ways in which mobile technology might help them and their patients and features they would want in such apps (e.g., *“How can mobile phones help in daily activities of the patient?”, “What should be the medium of contents in the mobile app?”, and more*). Finally, the FGDs ended with the moderators trying to capture the fear and apprehensions of caregivers in using mobile app based solutions.

### Data capture and analysis

The quantitative data was recorded in paper based questionnaires and was analyzed using STATA (ver12.0) statistical package. The questionnaire was administered in Hindi or English by a trained psychiatrist depending on the participant’s comfort and preference for the language. All the FGDs were conducted in Hindi, audio-recorded and subsequently transcribed and translated into English. Qualitative data was subjected to manual inductive thematic coding [55] by reading and simultaneously coding the transcripts line by line. The analysis was carried out by two researchers independently and comparison between codes to identify recurring themes were conducted by a third consultant. The examples of initial patterns that emerged through coding include—*“medication,” “daily challenges of caregiving,” “lack of information,” “stigma,” “support for caregivers,”* and more. The initial data was revisited until theme exhaustion was achieved and theme clustering into categories were done in subsequent analysis sessions. These categories were grouped into eight themes presented in the results section.

### Reflexivity

We are a team of medical professionals (Psychiatrists) and computer scientists. All lead psychiatrists for the study have experience of more than 10 years in the field, and are academic faculty who deal with patients and caregivers of people suffering from SMIs and training residents the scientific principles of management. Although Global West has seen the use of mobile based solutions in managing SMIs, our clinical experience makes us aware of the various challenges faced in adopting such technology based protocols. In this work, we dwell on our experience of dealing with such patients and their family caretakers to understand the opportunities and challenges of mobile application based interventions in managing SMIs in an Indian context. We have worked with a team of human computer interaction (HCI) researchers who have field experience of working with various minority population across different settings in India. The researchers have worked on several projects where they leveraged technology for social good with a keen focus in the domain of health and education.

## Results

### Findings from the survey

The questionnaire-based survey was completed by 88 patient-caregiver pairs (176 subjects), and an additional 9 incomplete response pairs were discarded due to difficulty in answering questions related to mobile technology. The important socio-demographic profile of patients and their caregivers along with the clinical details of the patients are provided in Table 2. A significant difference (t-test, p = 0.0021) was noted in the age of caregivers who possessed smartphones compared to those who did not in the bivariate analysis. The mean age of caregivers having smartphones was about a decade younger (age: mean = 41.14years, SD = 13.37) compared to those who did not have smartphones (age: mean = 49.18years, SD = 10.29). In contrast, no such difference was noted in patients’ use of smartphones in relation to their age (t-test, p = 0.864). The qualitative interviews revealed that many patients who previously owned smartphones switched to simple phones or no phones at all after the onset of the illness due to the fear of damaging such costly devices or due to the fear of misuse of technology, Internet and social media.

**Table 2:** Demographic characteristics of participants & clinical profile of patients of SMI: (n = 88).

Characteristics (values)^α^	Patients (n = 88)	Care-providers (n = 88)
**Age** mean (SD)	33.3 (10.8)	46.1 (12.2)
**Gender:** Male	54 (61.4)	37 (42.1)
**Marital Status:** Married	40 (45.5)	79 (89.8)
**Education**		
No formal education	2 (2.3)	10 (11.5)
Formal Education	51 (57.9)	53 (60.2)
Graduate	27 (30.7)	19 (21.8)
Post-graduate	8 (9.1)	6 (6.9)
**Occupation**		
Never worked	47(53.4)	1 (1.1)
Unskilled/skilled work	11 (12.5)	26 (29.5)
Professional	9 (10.2)	15 (17.1)
Housewife	10 (11.4)	36 (40.9)
Other (students /retired/farmers, etc)	11 (12.5)	10 (11.4)
**Currently Employed** *	20 (22.7)	47 (53.4)
**Diagnosis**		
Schizophrenia	30 (34.1)	—
Other psychosis	34 (38.6)	
Bipolar disorder	24(27.3)	
**Course of illness**		
Continuous	64 (72.7)	—
Episodic	24 (27.3)	
**Age of onset of illness** (years)	23.8 (8.2)	—
**Total duration of illness** (years) [median(IQR)]	7.5 (3.5, 14)	—
**Time in treatment** (years) [median(IQR)]	5(3, 10)	—

^*α*^ All values as n(%) unless specified otherwise.

* Employment is defined as an income earning job outside the home and excludes the occupational categories of home-maker and student.

#### Caregivers and time devoted to patient care activities

The majority of the families seeking treatment were urban Hindus (n = 78, 88.6%) belonging to the middle socio-economic class with the median monthly family income being around INR 25000 (US $384) albeit with wide variation (IQR: 15,000-40,000). The primary caregivers were mostly parents (mother: 28, 31.8%; father: 22, 25.0%) or spouses (wife: 20, 22.7%; husband: 8, 9.1%). Children seldom identified themselves as the primary care provider (Son: 6, 6.8%; Daughter: 0, 0%). The majority of the patients suffered from schizophrenia and related psychoses (n = 64, 72.7%).

Caregivers spent a considerable proportion of their day (mean: 3.85 hours, SD: 3.88) in caregiving activities. The commonest care giving activity reported was the need to supervise medication (n = 28, 31.8%) and the supervision of basic self-care of patients (n = 26, 29.5%) in terms of getting up in time, brushing, bathing etc. A significant number of patients required coaxing and support of all activities (n = 13, 14.8%) or general non-specific supervision (n = 8, 9.0%). Other specific care-giving responsibilities included bringing the patient to hospital (n = 8, 9.0%), supervising studies (n = 2, 2.2%) and taking patient out of the house (n = 1, 1.1%).

#### Mobile technology penetration and health technology use

In our data, the rate of smartphone ownership was similar to the national average in both care providers (38.6%) and in patients (31.8%). Most caregivers and patients who had smartphones were aware of their phones’ operating systems and used common apps (Facebook, WhatsApp) regularly. However, only a few of them knew about the sensor capabilities of their phones and they rarely downloaded health apps (Table 3). Most care providers reported the perceived need for information regarding the management of their patients, although actual usage of freely available health apps was much less. The majority of respondents viewed such MHapps as helpful, while 13 respondents considered such apps to be an additional burden for them to use. The details of caregiver’s health technology usage and attitudes towards mobile based mental health solutions are provided in Table 4.

**Table 3:** Mobile technology usage of patients and caregivers of SMI: (n = 88).

Characteristics (values)	Patients (n = 88)	Care-providers (n = 88)
**Phones ownership:** n(%)		
No Phone	11 (12.5)	3 (3.4)
Simple Phone	49 (55.7)	51 (57.9)
Smartphone	34 (38.6)	28 (31.8)
**Smart-phone software platform:** n/N*(%)		
Android	20/28 (71.4)	26/34 (76.5)
iOS	1/28 (3.6)	1/34 (2.9)
Windows	1/28 (3.6)	1/34 (2.9)
Unaware	6/28 (21.4)	6/34 (17.7)
**Smartphone technology usage:** n/N*(%)		
Internet	23/28 (82.1)	27/34 (79.4)
WhatsApp	25/28 (89.3)	21/34 (61.8)
Facebook	23/28 (82.1)	21/34 (61.8)
Games	20/28 (71.4)	15/34 (44.1)
**Aware of phone sensors:** n/N*(%)		
Camera	56/77 (72.7)	55/85 (64.7)
GPS	16/28 (57.1)	18/34 (52.9)
Accelerometer	5/28 (17.9)	8/34 (23.6)
HR Sensor	4/28 (14.3)	6/34 (17.7)

* N = Number of persons with relevant phone type.

**Table 4:** Usage and attitude towards mobile based health technologies in caregivers of SMI: (n = 88).

Parameters	Values: n/N*(%)
Has access to internet	28/88 (31.8)
Has access to internet over landline phone	14/88 (15.9)
Has access to internet over mobile phone	27/88 (30.7)
Downloads apps on smartphone	24/34 (70.6)
Number of apps downloaded in past month: median (Range)	2 (0-10)
Number of health apps downloaded ever: median (Range)	0 (0-3)
Used a smartphone to access health information	19/34 (55.9)
Used a smartphone to access health services (appointments/test results)	16/34 (47.1)
Wants access to information related to patient’s illness via **text messages**.	83/88 (94.3)
Wants access to information related to patient’s illness via **MHapps**.	57/88 (64.8)
Willing to **download** an “app” to phone to help monitor the patient’s illness.	57/88 (64.8)
Willing to use the “app” on a **daily** basis to monitor patient’s health.	55/88 (62.5)
Thinks such “app” will be **helpful** in the various caregiving activities.	54/88 (61.4)
Think such“apps” will add to the **burden** of caregiving.	13/88 (14.8)

* N = Number of persons with relevant phone type.

### Findings from the focus group discussions (FGDs)

The major themes emerging from the FGDs are summarized in Table 5. We dwell on these themes to present the detailed inferences in this section. In many cases, the findings of the FGDs supplement and elaborate on the results obtained from the quantitative survey.

**Table 5:** Iterative inductive thematic analysis of three FGDs resulted in eight major themes. The table presents the themes, each supported by an example quote from the data set. To present our inferences in the findings section, we further group these themes into three categories: (1) scope of mobile apps in improving patient-care; (2) scope of mobile apps in empowering caregivers; and (3) caregivers’ apprehensions about using mobile based apps for healthcare.

Theme	Definition	Example (Quotes from FGDs)
Medication Adherence	Patient’s reluctance to take medication and possible methods to promote medication adherence.	*“Patients could provide the doctors with false feedback [about medicine intake] using the acknowledge button to get rid of medication adherence” [F1C4]*.
Tracking Health Improvement	Cases where tracking patient’s health would prove to be helpful and beneficial (e.g., impact of the treatment).	*“If I get the information about what all activities of daily living like brushing, bathing he has done today, then it will fill my heart with a sense of satisfaction and peace that he has improved” [F3C1]*.
Connectivity with Doctors	The need for a ubiquitous communication link with the doctors/experts.	*“Like [a] call center where one can record and drop a question and later an expert could provide us with a solution via call” [F1C1]*.
Act of Caregiving	Various challenges of caregiving and strategies devised to cope with the given challenges.	*“If we want them to do something, then we have to ask them repeatedly. If they do not take medicine, then our 100% time is invested [in persuading] them to wake up, take bath, eat food. Sometimes we even lure them like –get up, get ready then we would go shopping” [F2C2]*.
Empower the caregiver	Need for support in caregiving including authentic information, emotional support, and more.	*“What kind of atmosphere should the patient be provided with when back at home, we would like to know about all this using the given platform” [F3C3]*.
Stigma	Societal stigma associated with mental illnesses and how it affects the daily lives of patients and their families.	*“Psychiatric patients simultaneously begin shouting, beating, assaulting. Now, mostly, people don’t know about mental illness, so when they see such behavior, at first place they say that the person is in captivation of a spirit, it ‘s a ghost, they don’t approach the doctor” [F2C3]*
Affordability, Availability, & Access	Various practical challenges pertaining to affordability, access, and availability of the required solution.	*“Internet is good as well as bad, if I use it for a good purpose then the result will be good, but if I use it for the wrong purpose then the outcome will be bad…if I provide the patient with Internet access and let him use it as per his convenience then there are higher chances of a negative outcome” [F3C1]*.
Literacy	Challenges of local vernaculars, proficiency with English and digital illiteracy.	*“We can do plenty of things via mobile, but one should know how to operate it, right?!” [F3C5]*.

#### Scope of mobile apps in improving patient care

Mobile apps were most commonly viewed as a tool to provide information to patients and their family members. Information regarding the nature and course of illness, the treatment and management of side effects, vocational rehabilitation, emergency services, government benefits and schemes, ambulance, daycare centers, and NGOs were highly sought after. Although such information is available on the internet, many caregivers wanted information from sources they could trust. Internet results on mental disorders and its treatment were often in conflict, with many websites focusing on the benefits of not taking any treatment or highlighting the side effects of medicines, which patients often selectively focused on. Information from treating doctors often helped family members in counseling patients to continue treatment. The caregivers wanted apps that would help monitor patients’ compliance to medication and daily self-care tasks, thereby decreasing the load on caregivers. Alarms and reminders [F1C1] on both patients’ and caregivers’ phones, with a digital diary [F1C3] to monitor such tasks was considered welcome. Caregivers preferred individualized and flexible apps, which take into consideration the time-table of the patient and the day to day variations in family life. Apps that provided patients with a menu of choices for tasks, from which they could select a few per day [F3T1] were considered to be more compliable than a rigid schedule. Dwelling on experiential knowledge, one of the caregiver said:

“These patients do not perform activity of daily living normally, and if we bind them to a tight schedule using the application, they will not do it anyway”

[F3C4].

Furthermore, passive monitoring of patient activity [F3C1] without family member intervention was also considered a welcome help.

Participants also suggested the use of apps to provide motivating messages, stories and poems to improve the mood of the patient. Two caregivers reported use of mobile phones to keep patients involved while they completed household chores. Engaging the patient in mobile games often decreased aggression and distress in patients and music often helped patients relax. By their experience, caregivers suggested that compliance to medications and involvement in daily activities could be improved by gamification of targets or by even opening a level in a mobile game as a reward. Apps which provide information regarding the possible side effects of medications [F1C2] and the assurance that these side effects are resolvable even before they actually occur can also improve adherence [F3R1]. Most participants wanted health information to be provided through audiovisual means, as reading text was often difficult for many of these patients. One participant proposed a novel approach for patients to track their improvement by recording short duration self-videos. As improvement in severe mental disorders is very gradual, family members and patients tend to get demoralized. Recording a short video clip about self fortnightly and comparing them over time could make patients aware of their improvement and hence more compliant:

“If a patient can upload a self-video about how he is feeling, about once in a week, then the patient can be shown ‘Look, this is what happened’..”

[F1C3].

Such success videos, if shared can then also provide other patients and family members with hope.

#### Scope of mobile apps in empowering caregivers

Most caregivers responded that patient-care consumed significant amounts of time and effort. During periods of illness, patients needed support and supervision for all 24 hours, while during remission a significant amount of time, about 4-5 hours per day [F3C1, F3C2, F3C3] was spent on taking care of the patient. Making patients perform simple daily chores and activities was a difficult task as patients themselves preferred to remain inactive and would often become irritable and recalcitrant. Arguments, resentments, and stress between caregivers and patients were common in such situations, and caregivers often felt helpless, particularly if the patient was older than the careprovider[F1C3]. With chronic resistance from patients, caregivers often suffered burnout and accepted many goals as unattainable to their patients. Patient care was generally provided by one key family member, as others gradually became indifferent to or critical of the patient [F3C2]. According to the caregivers, the patient’s response varied with the patient’s own perception of a task’s importance. When such activities were suggested by doctors or senior family members, patients were more likely to perform them.

Most participants wanted mobile apps to be provided to the caregiver in addition to the patient. While many wanted the same app for the patient and caregiver, some wanted the caregiver’s app to contain more information regarding management of patients, as such information was not readily available on the internet:

“Patient-related information is available on the Internet, but I have spent nights after nights in search of information on how a caregiver can handle patient[s] in certain situation, there is no data available for us”

[F1C3].

“Up till now, we people do not have enough knowledge about mental illness. From my understanding, 70% of people in India do not know; we also didn’t know; it was only after coming here that we came to know about so many types of mental illnesses that prevail with different symptoms and conditions…so if you provide information in this regard on the phones, it would be really great”

[F2C4].

The lack of awareness on mental health and mental illnesses has led to stigmatization of the subject. For example, F3C2 said,

“[If] there is [a] problem related to [the] heart or, say, lungs, people will go rushing to doctors for the treatment. [The] problem is that nobody treats mental illness as a form of illness, especially here in India; rather, it is associated with spirits, ghosts or religious belief[s].”

The caregivers emphasized the need for an accessible and authentic source of information on the topic, which can be used to create awareness on the subject:

“The misconception of people that the child has gone mad needs to be rectified…If [by] somehow using this medium, we could convince people that this is not madness, it can be treated, and it’s temporary, this is no big deal… so that the kid also doesn’t get affected by such comments”

[F2C6].

All the participants considered vernacular language translation of the app essential as English was often difficult to understand:

“If you talk about village level, then there is [a] high possibility that nobody knows English”

[F3C1].

Furthermore, three participants suggested the creation of a caregiver social network through a mobile app, so that support and motivation can be provided within the group without needing a doctor’s intervention. Moreover, such an app might also be beneficial to caregivers when they step away from their patients. The participants who did not have smart phones requested simple alternatives for information delivery like SMS or IVRS (Interactive Voice Response System) which they were comfortable operating. There was a perceived need for an app to detect caregiver burnout, diagnose depression, and anxiety in caregivers and provide stress management for them, although it was not under the purview of the current research.

#### Caregiver’s apprehensions in using mobile based apps for health care

Family members were apprehensive that patients might damage a *“costly smart phone”* due to their illness. According to most caregivers, lack of money either to own a smartphone or an active Internet connection was a major problem. Lack of proficiency in understanding English emerged as another major barrier as most Internet based apps and resources were available in the English language. The reliability and the consistency of the health app was mentioned as an important consideration as patients lose interest in unreliable apps very quickly. Furthermore, the caregivers said that the treating doctor is needed to promote an active positive perception towards the app. If treating clinicians do not emphasize the importance of such apps, patients and family members are generally discouraged from using them. Many caregivers reported that learning a new app was challenging for them and therefore, a period of training for both patient and caregiver was required for the success of any such endeavor. One participant [F2C5] was concerned about the *“overuse of mobile phones”* by the patients in the pretext of using the app. Similarly, caregivers raised their concern over *“Internet addiction”* and *“game abuse”* which might prevent the patient from following a structured daily routine. Meanwhile, one caregiver [F2C4] opined that the unsupervised use of the Internet may be harmful to the patient as there were many inappropriate topics over the Internet. Family members were also worried that patients might place unwanted calls to strangers or relatives through mobile phones. Caregivers reported that due to such concerns, many have actually changed the smart-phone that patients used before the onset of illness to simpler handsets.

## Discussion

Existing work on SMIs like bipolar disorder [56–58] and schizophrenia [31, 59–61] demonstrate the feasibility, acceptability, and utility of smartphone based interventions, albeit in resource rich settings. Further, currently available mHealth applications, which perform participatory and unobtrusive data sensing to assist the patient in self-monitoring, focus on patient-centric mobile interventions but neglect the role of family members as care providers, a hallmark of the Indian collectivistic family system. Our study provides a primer on the perceived needs of caregivers of people suffering from SMIs in India, insights about their (both the patients’ and the caregivers’) smartphone usage patterns concerning healthcare needs/services, and the practical roadblocks faced in using smartphone based interventions.

We observed that the smartphone possession and usage in patients and caregivers of SMI closely follow the national average of around 30% [53], suggesting a definite scope for deploying smartphone based solutions. However a significant 55-58% of patients and caregivers had access only to regular phones and 68% of caregivers had no access to the Internet, suggesting a strong need for a *pluralistic* approach where we develop non-internet based parallel solutions for wider reach. For example, Interactive Voice Response Systems (IVRS) based solutions, as deployed for HIV/AIDS, might provide an interim viable solution for the country in a resource constrained setting [62]. Our data also revealed that many caregivers switched from smartphones to basic phones to avoid the negative influence of unsupervised internet access on the patients. Also, not all patients follow the same routine or indulge in the same activities. This calls for a feature of *customization* as per the choice of individual users.

The results of the FGDs highlighted the huge investment of time and effort taken on by caregivers, often to the exclusion of their own needs. The reintegration of such patients into society requires continuous encouragement and effort from family members, burdening them enormously and leading to caregiver burnout [63]. The family’s potential in facilitating recovery from an illness has generally been unrecognized, and most studies are patient centric with an aim to provide effective care, neglecting the caregiver’s wellbeing and needs [64]. Although mental health professionals in India welcome the family caregiver’s involvement, little has been done to reduce the caregiver’s burden [65]. This demands an *ecological* design approach where we tailor existing mHealth interventions in accordance with the Indian context to accommodate the harmony shared by patients and their caregivers in coping with SMIs.

Our survey data demonstrated that although caregivers wanted health technology solutions to their problems, few, if any, had searched for such free MHApps on the Internet or were using them. Limitations in understanding the English language and low general technology literacy surfaced as factors which prevent the use of MHApps in patients and caregivers, suggesting the need for increased handholding in such groups. The concerns related to language of communication are understandable in a country like India, which is home to different vernaculars. Although India stands second among the English speaking nations, only 10% of the Indian population falls in the category using English as a second or third language [66]. A similar trend can be observed in Internet usage statistics report that states that 37% (45 million) of all active Internet users (including 64% from rural India and 25% of urban India) access the Internet in their regional language [37]. The existing gap between the desire for a technology based solution and its adoption highlights the importance of *participation* of stakeholders (caregivers in this case) in the technology design process. The importance of *participatory design* was reinforced when caregivers revealed false data input by the patients as one of the major impedance in the success of MHApp adoption. A similar effect was observed by Joshi et al.Â [62] on people suffering from HIV/AIDS where six out of the fifty participants faked dosage intake, while two faked symptoms. This result shows that a motivated user may cheat the system despite knowing that it could affect their illness’s course for various reasons. Thus, caregivers suggested that providing an app for the caregiver and including caregiver’s feedback into the loop, will additionally help in reliability of the monitoring data generated out of any such app system.

Another factor that impacts the adoption of any MHApp based intervention is the authenticity of the source of information and acceptance of and promotion by treating clinicians. Thus, the ecological design approach should use a *participatory design* process where we engage all the primary stakeholders (e.g., patients, caregivers and clinicians) as designers in the project.

Moreover, mental disorders are subjected to increased stigma [67], which makes seeking help difficult for patients and their family members. As researchers we need to provide information that can create awareness about mental health issues for the general public. Some caregivers expressed that they needed information to show to people in order to convince them that mental health is only a health issue just like blood pressure or diabetes. Stigma was a recurring theme in discussions, and thus, there is a need to provide psycho-social education that not only makes the user knowledgeable but also *advocates* for a better understanding of mental health issues.

Through our study, we learned that a *pluralistic* and *ecological* approach to design where various stakeholders *participate* as designers is required for the successful adoption of MHApps in India. This approach can also be generalized for other resource constrained (infrastructure, network issues, literacy and more) low and middle income countries with the same challenges, but researchers should note that stakeholders may vary across the countries, depending upon the cultural context and societal practices.

## Limitations

This paper describes our initial effort to understand the MHApp scope for severe mental disorders in India by using quantitative and qualitative design strategies. It suffers from certain short-comings. First, a tertiary care center caters to more chronic and severe cases, so the findings might be difficult to generalize to other primary care centers. Second, although our survey looked into smartphone usage, it was not possible to verify participants technology skills objectively. Further, mixed group FGD with caregivers and service providers could have inhibited some participants from coming out with their problems although such mixed groups were deliberately formed to understand contrasting perspectives.

Despite the limitations, our study is one of the first from our country that systematically looks into the scope, design considerations, and limitations of implementing a mobile technology based intervention. The recent intense interest in mobile based health apps suggests a definite niche for such interventions in bridging the MHGap. Simultaneously, most reviews also highlight the inherent problems of such interventions in their lack of comparability, non-availability, proprietary implementation, lack of generalizability, and undetermined long term effectiveness. Our exploration of caregivers’ perspectives involved in managing mental illness, and technology experts involved in developing such solutions, provides a guiding framework for developing technology enabled solutions for interventions in severe mental disorders.

## Supporting information

S1 File SurveyFormHindi. This file includes Hindi version of the survey used for the study.
(PDF)

S2 File SurveyFormEnglish. This file includes English version of the survey used for the study.
(PDF)

S3 File SurveyResponse. This file includes all the survey responses received and used in analysis for the study.
(XLSX)
