# Supplementary material for: Is India ready for mental health apps (MHApps)? A quantitative-qualitative exploration of caregivers’ perspective on smartphone-based solutions for managing severe mental illnesses in low resource settings
Source: PLoS One. 2018 Sep 19;13(9):e0203353. doi: 10.1371/journal.pone.0203353 (PMC6145572; doi:10.1371/journal.pone.0203353)
Supplement: S1 File — This file includes Hindi version of the survey used for the study. (PDF) [file pone.0203353.s001.pdf]

कम - संसाधन वाले देशों में, गंभीर मानसिक बीमारियों से पीड़ित मरीज़ों और उनके परिवार में, मोबाइल-आधारित हस्तक्षेप की ज़रूरतों को समझना

देखभालकर्ताओं की जानकारी:

**मरीज़ से सम्बन्ध:**	पिता	माँ	बेटा	बेटी	रिश्तेदार (स्पष्ट करें)		
**उम्र (साल में)**		केंद्र से दूरी (किलोमीटर)					
**लिंग**	पुरुष	महिला	अन्य				
**वैवाहिक स्थिति**	अविवाहित	विवाहित	अलग रहते हैं	तलाकशुदा	विधवा / विधुर	अन्य	
**शिक्षा**	अनपढ़	पढ़े - लिखे	प्राथमिक शिक्षा	मध्य - शिक्षा	स्नातक	स्नातकोत्तर	
	अन्य :				शिक्षा के कुल वर्ष:		
**व्यवसाय**	बेरोज़गार	अकुशल कर्मचारी	कुशल कर्मचारी	पेशेवर	गृहणी	सेवानिवृत्त	छात्र
	व्यवसाय	किसान	अन्य :				
**वर्तमान में रोज़गार की स्थिति**	हाँ	नहीं	आंशिक रूप से		पिछले ३० दिनों में रोज़गार की स्थिति		
**मानसिक आमदनी**					कुल पारिवारिक आय स्पष्ट करें		
**धर्म**	हिन्दू	।मुस्लिम	ईसाई	सिख	अन्य		
**देखभाल में व्यतीत समय**					घंटे/दिन में बताएं की मरीज़ की देखभाल में कितना समय लगता हैं		
**सारी सामान्य गतिविधियां जिसमे मरीज़ को मदद देनी पड़ती है**	1)						
	2)						
	3)						

मरीज़ की जानकारी:

**आई सी डी निदान**					फाइल के मुताबिक़:		
**उम्र (साल में)**							
**लिंग**	पुरुष	महिला	अन्य				
**वैवाहिक स्थिति**	अविवाहित	विवाहित	अलग रहते हैं	तलाकशुदा	विधवा / विधुर	अन्य	
**शिक्षा**	अनपढ़	पढ़े - लिखे	प्राथमिक शिक्षा	मध्य - शिक्षा	स्नातक	स्नातकोत्तर	
	अन्य :				Total years of education		
**व्यवसाय**	बेरोज़गार	अकुशल कर्मचारी	कुशल कर्मचारी	पेशेवर	गृहणी	सेवानिवृत्त	छात्र
	व्यवसाय	किसान	अन्य				
**वर्तमान में रोज़गार की स्थिति**	हाँ	नहीं	आंशिक रूप से		पिछले ३० दिनों में रोज़गार की स्थिति		
**बीमारी शुरू होने की उम्र**			**बीमारी की कुल अवधि**				
**बीमारी का कोर्स**			**इलाज की कुल अवधि**				
**प्रधान उपचार**	एंटी सायकोटिक	एंटी डेप्रेस्सेंट	मूड स्टेबलाइजर				
**प्रधान दवाई का नाम**							
**परिवार में मानसिक बिमारी की जानकारी**					परिवार में किसी को मानसिक बिमारी है, तो उसके बारे में बताये		

कम - संसाधन वाले देशों में, गंभीर मानसिक बीमारियों से पीड़ित मरीज़ों और उनके परिवार में, मोबाइल-आधारित हस्तक्षेप की ज़रूरतों को समझना

मोबाइल फ़ोन और इंटरनेट की आदतें और ज़रूरतों से सम्बंधित जानकारी

**भाग ए : मोबाइल फ़ोन का इस्तेमाल**					
क्या आपके पास वर्तमान में मोबाइल फ़ोन है?	हाँ	नहीं	**फ़ोन मोडल नंबर:**		
वह किस तरह का मोबाइल फ़ोन है?	साधारण फ़ोन	स्मार्ट फ़ोन			
आपका फ़ोन किस प्लेटफार्म का उपयोग करता है?	एंड्रॉइड	आई ओ एस	विंडोज	अन्य	
आपके फ़ोन में कौन-कौन से सैंसर हैं?	कैमरा	जी पी एस	एक्सेलेरोमीटर	एच आर सेंसर	अन्य
ज़्यादातर आप अपने फ़ोन का इस्तेमाल किन चीज़ों के लिए करते हैं?	बातचीत	एस एम एस	इंटरनेट	ईमेल	खरीदारी
	व्हाट्सऍप	फेसबुक	खेल	अलार्म	कैलेंडर
क्या आपके मरीज़ के पास मोबाइल फ़ोन है?	हाँ	नहीं	**फ़ोन मोडल नंबर:**		
वह किस तरह का मोबाइल फ़ोन है?	साधारण फ़ोन	स्मार्ट फ़ोन			
उनका फ़ोन किस प्लेटफार्म का उपयोग करता है?	एंड्रॉइड	आई ओ एस	विंडोज	अन्य	
उनके फ़ोन में कौन-कौन से सैंसर हैं?	कैमरा	जी पी एस	एक्सेलेरोमीटर	एच आर सेंसर	अन्य
ज़्यादातर आपका मरीज़ अपने फ़ोन का इस्तेमाल किन चीज़ों के लिए करता है?	बातचीत	एस एम एस	इंटरनेट	ईमेल	खरीदारी
	व्हाट्सऍप	फेसबुक	खेल	अलार्म	कैलेंडर
**भाग बी: इंटरनेट-सम्बन्धी व्यवहार**					
क्या आपके पास इंटरनेट का साधन है?	नहीं	हाँ	लैंडलाइन	मोबाइल	
क्या आपके फ़ोन में "एप्स"/ आप्लिकेशन्स डाउनलोड हो सकती हैं?	नहीं	हाँ	आपके फ़ोन में कितनी एप्स हैं?		
आप हर महीने अपने फ़ोन में कितनी एप्स डालते हैं?		आपके फ़ोन में स्वास्थ-सम्बन्धी कितनी एप्स हैं?			
**पिछले ६ महीनों में क्या आपने अपना स्मार्ट फ़ोन इन गतिविधियों के लिए इस्तेमाल किया है?**<br>- सामान्य स्वास्थ-सम्बन्धी जानकारी लेने के लिए?		हाँ (खुद के लिए)	नहीं (खुद के लिए)	हाँ (मरीज़ के लिए)	नहीं (मरीज़ के लिए)
- अपनी व्यक्तिगत स्वास्थ्य देखभाल जानकारी जैसे परीक्षण परिणामों या नियुक्तियों को निर्धारित करने के लिए		हाँ (खुद के लिए)	नहीं (खुद के लिए)	हाँ (मरीज़ के लिए)	नहीं (मरीज़ के लिए)
- क्या आप अपने स्मार्टफोन के माध्यम से अपने रोगी की बीमारी से संबंधित सामान्य जानकारी तक पहुंचने में सक्षम होना चाहते हैं?				हाँ	नहीं
-क्या आप अपने डॉक्टर से अपने मरीज के स्वास्थ्य से संबंधित टेक्स्ट संदेश प्राप्त करना चाहते हैं?				हाँ	नहीं
-क्या आप अपने स्मार्टफोन पर "ऐप" के माध्यम से अपने रोगी की चिकित्सा-सम्बंधित स्थिति को ट्रैक करने में मदद चाहते हैं?				हाँ	नहीं
-क्या आप अपने मरीज की स्वास्थ्य स्थिति की निगरानी में मदद के लिए अपने फोन पर "ऐप" डाउनलोड करेंगे?				हाँ	नहीं
-क्या आप, रोज़मर्रा मैं, ऐप के जरिये अपने स्वास्थ की निगरानी रखना चाहेंगे?				हाँ	नहीं
-क्या आप सोचते हैं की इस तरह के ऐप से आपको अपने रोगी की देखभाल करने में मदद करेगी?				हाँ	नहीं
-क्या आप सोचते हैं की इस तरह की ऐप रोगी की देखभाल के बोझ को और भड़ा देगी?				हाँ	नहीं

-मोबाइल फ़ोन और इंटरनेट का इस्तेमाल करके किस तरह की देखभाल-सम्बन्धी गतिविधि में मदद मिलेगी? (एक गतिविधि बताएं)

---ख़त्म---
