# Supplementary material for: Is India ready for mental health apps (MHApps)? A quantitative-qualitative exploration of caregivers’ perspective on smartphone-based solutions for managing severe mental illnesses in low resource settings
Source: PLoS One. 2018 Sep 19;13(9):e0203353. doi: 10.1371/journal.pone.0203353 (PMC6145572; doi:10.1371/journal.pone.0203353)
Supplement: S2 File — This file includes English version of the survey used for the study. (PDF) [file pone.0203353.s002.pdf]

Understanding caregiver & patient needs for mobile based intervention in severe mental illnesses in low-resource settings

Performa for the CAREGIVER:

**Relation to patient**	Father	Mother	Son	Daughter	Relative (Specify)		
**Age** (years)		**Distance from Center** (km)					
**Gender**	Male	Female	Other				
**Marital Status**	Never Married	Married	Separated	Divorced	Widowed	Other:	
**Education**	Illiterate	Literate	Primary	Middle	Graduate	Post-Graduate	
	Other:				Total years of education		
**Occupation**	Unemployed	Unskilled worker	Skilled Worker	Professional	Housewife	Retired	Student
	Business	Farmer	Other:				
**Current Employment**	Yes	No	Partly		Specify employment status in last 30 days		
**Monthly Income**					Specify total family income in INR		
**Religion**	Hindu	Islam	Christianity	Sikhism	Other:		
**Time spent in caregiving**					Specify, time in hours/day, that are needed for caregiving of the patient		
**Common activities requiring support**	1)						
	2)						
	3)						

Performa for the PATIENT/WARD of the caregiver:

**ICD Diagnosis**					As per file		
**Age**					In years		
**Gender**	Male	Female	Other				
**Marital Status**	Never Married	Married	Separated	Divorced	Widowed	Other:	
**Education**	Illiterate	Literate	Primary	Middle	Graduate	Post-Graduate	
	Other:				Total years of education		
**Occupation**	Unemployed	Unskilled worker	Skilled Worker	Professional	Housewife	Retired	Student
	Business	Farmer	Other:				
**Current Employment**	Yes	No	Partly		Specify employment status in last 30 days		
**Age of onset of illness**			**Total Duration of Illness**				
**Course of illness**			**Time in treatment**				
**Principal Treatment**	Antipsychotic	Antidepressants	Mood Stabilizers				
**Principal Medication Name**							
**Family History**					Specify history of psychiatric disorders in 1^0 relatives		

Understanding caregiver & patient needs for mobile based intervention in severe mental illnesses in low-resource settings

Mobile Phone and Internet Use Habits & Needs Questionnaire

**Part A: Mobile Phone Use**					
Do you currently own a mobile phone?	Yes	No	**Phone Model No:**		
What type of mobile phone is it?	Simple Phone	Smart Phone			
What platform does your phone operate on	Android	iOS	Windows	Other:	
What sensors do your phone have	Camera	GPS	Accelerometer	HR Sensor	Other:
What do you generally do with your phone	Call	SMS	Internet	email	Shop
	WhatsApp	Facebook	Games	Alarm	Calendar
*Do your **patient** own a mobile phone?*	Yes	No	**Phone Model No:**		
What type of mobile phone is it?	Simple Phone	Smart Phone			
What platform does his phone operate on	Android	iOS	Windows	Nokia	Samsung
What sensors do the phone have	Camera	GPS	Accelerometer	HR Sensor	Other:
What does your ward generally do with the phone	Call	SMS	Internet	email	Shop
	WhatsApp	Facebook	Games	Alarm	Calendar
**Part B: Internet related Behavior**					
Do you have access to internet	No	Yes	Landline	Mobile	
Can your phone download applications or "apps"?	No	Yes	How many apps on phone		
How many "apps" do you put on your phone each month?		How many health care–related "apps" do you have on your phone?			
**In the past 6 months, have you used your smartphone**<br>- to access general health care information?		Yes (for self)	No (for self)	Yes (for patient)	No (for patient)
- to access your personal health care information such as, test results or to schedule appointments?		Yes (for self)	No (for self)	Yes (for patient)	No (for patient)
Would you want to be able to access general information related to your patient's illness via your smartphone?				Yes	No
Would you want to receive text messages related to your patient's health from your doctor's office?				Yes	No
Would you want to use your phone to help track your patient's medical condition via an "app" on your smartphone?				Yes	No
Would you download an "app" to your phone to help monitor your patient's health condition?				Yes	No
Would you be willing to use an application or "app" on your phone on a **daily** basis to help monitor your health condition?				Yes	No
Do you think such "app" will be helpful in the various caregiving activities that you do?				Yes	No
Do you think such "apps" will add to the burden of caregiving?				Yes	No
What caregiving activity can be helped **most** by using mobile phones and internet? (Give a single activity)					

---END---
